# Older Parents’ Cynical Hostility and Their Relationships with Their Adult Children: A Longitudinal Dyadic Study of North American Couples

**DOI:** 10.3390/healthcare11050736

**Published:** 2023-03-02

**Authors:** Dikla Segel-Karpas

**Affiliations:** Department of Gerontology, University of Haifa, Haifa 3498838, Israel; dsegel@univ.haifa.ac.il

**Keywords:** cynical hostility, intergenerational relationships, older parents, social relationships

## Abstract

Older adults’ relationships with their children are often a source of reciprocal emotional and instrumental support, but also of strain. Cynical hostility is a cognitive schema, according to which people cannot be trusted. Previous studies showed that cynical hostility has adverse implications for social relationships. Little is known about the possible outcomes of parental cynical hostility on older adults’ relationships with their children. Two waves of the Health and Retirement Study and Actor–Partner Interdependence Models were used to examine the way spouses’ cynical hostility at Time 1 is associated with their own and their spouse’s relationship with the children at Time 2. Both partners’ cynical hostility predicts his or her own strain in the relationship with the children, and for husbands, their spouse’s cynical hostility also predicts strain. For husbands only, their own cynical hostility is associated with reduced perceived support from their children. Finally, a husband’s cynical hostility is associated with both partners’ reduced contact with their children. These findings illuminate the social and familial costs of cynical hostility in old age, suggesting that older adults with higher levels of cynical hostility may be more susceptible to strained relationships with their children.

## 1. Introduction

Family relationships in general, and the parent–child relationship in particular, are one of the most important relationships throughout life, and a source of support for both older parents and adult children [1,2]. However, while some older adults enjoy satisfying and meaningful relationships with their adult children, others report that their relationships are ambivalent or conflictual, where interactions result in strain or disappointment [3]. Understanding what factors contribute to better parent–child relationship in adults is of interest to both scholars of family relations and practitioners. From a theoretical standpoint, according to Bowen [4] family dynamics are transmitted between individuals and through generations. Hence, depicting factors that shape the adult child’s relations with his or her parents can highlight the potential consequences of the way in which processes unfold in the family’s developmental trajectory and shape the relations between multiple generations and actors. Practically, the identification of factors that risk good parent–adult child relations can help detect older populations at risk for poorer care provision within the family, and further stressors on their daily lives that can negatively affect their physical and mental health [3]. This study was focused on parents’ cynical hostility and examined the manner in which it shapes the perceived parent–child relationship.

Hostility, anger, and aggression are frequently examined together. Whereas anger is an emotion and aggression is a behavioral tendency, hostility describes a cognitive aspect, defined as an “attitude toward others, consisting of enmity, denigration, and ill will” [5] (p. 26). Cynical hostility (or cynicism) is a component of hostility, ascribing selfish motives to others’ actions and implying a lack of trust in others [5,6]. Those having higher levels of cynical hostility were found to experience greater loneliness [7], less support [8,9], more disrespect [10], and strain in social relationships [9]. Focusing on married older couples, Segel-Karpas and Ermer [9] found that a wife’s cynical hostility was a significant predictor of her own and her husband’s loneliness. This suggests that both individuals’ cynical hostility has implications for their spouse’s sense of adequate social ties, and, possibly, for the domestic atmosphere.

Given the importance of cynical hostility for individuals’ ability to preserve satisfying social relations and given the significance of parent–adult child relations for the health and well-being of both parents and children [11,12], it is important to understand the manner in which this social-cognitive schema shapes within-family relations. Older parents with higher levels of cynical hostility might be in double or even triple jeopardy: they may have not only dissatisfying relations with their greater social network, but also more strained family relations and less emotional support [13], which might leave them with fewer potential care providers. As cynical hostility is a major risk factor for varied health problems [14,15,16], highly cynical hostile older parents may need assistance at an earlier age and with greater probability than older parents with lower levels of cynical hostility.

In this study, it was hypothesized that the cynical hostility of both partners is related to their relationships with their children. Using a longitudinal dataset of older adults and their spouses and the Actor–Partner Interdependence Model (APIM), the study examined the manner in which a husband and wife’s cynical hostility predicts his or her own and marital partner’s support, strain, and contact in the relationship with their children.

### 1.1. Older Parent–Adult Child Relationship

The present increased life expectancy implies that intergenerational relationships often stretch over several decades, and, despite the normative expectation for “launching” the adult child into independence [3], the parent–child bond often remains close and is considered a most emotionally meaningful relation [17]. Adult children often become a source of both emotional and instrumental support [18,19] for their older parents, and their assistance is especially salient when the parents are coping with illnesses, functional decline, and disability. At the same time, older adults often provide support to their adult children [18], ranging from babysitting the grandchildren to letting adult children return home because of financial difficulties, despite the toll it takes on their quality of life [19].

The existing research on relationships between older parents and their adult children has focused mostly on either demographic factors, such as age, gender, or parental physical needs, or relational factors. However, as can be implied from the literature concerning younger parent–child relations and from the existing research on adult children [3], the parents’ personality (together with the child’s temperament and environmental stressors and strains) [20] can significantly affect the development of both the child and the relationships. For example, drawing from the interpersonal perspective on personality [21,22], parents’ predispositions, such as cynical hostility, can shape the marital relations, as their expectations from others can encourage a response that is in line with their previous expectations. Enmity between parents can spill over onto the parenting and parent–child relations [23]. Thus, parents’ traits can not only make current interaction more or less pleasant, but also shape the family history, loading current relations with more positive or negative emotions.

In this study, the role played by both marital partners’ cynical hostility and its effects on parent–adult child relationships was examined. Both quantitative (i.e., contact frequency) and qualitative (i.e., strain and support) aspects of the relationship were investigated, differentiating between mothers and fathers.

### 1.2. Cynical Hostility

Cynical hostility is a trait-like social–cognitive schema according to which people are driven by selfish motives with little to no regard for others. Hence, according to the schema, others cannot be trusted [5]. Studies on cynical hostility in older adulthood have focused mainly on its adverse physical outcomes, first and foremost, its effect on cardiovascular health [24]. However, cynical hostility is also a meaningful driving force shaping individuals’ social relationships. The basic belief that people are not to be trusted can deter those with a higher level of cynical hostility from establishing intimate and satisfying social relationships.

Several studies support this notion, finding that cynical hostility adversely affects social relationships. In a cross-lagged panel model, Segel-Karpas and Ayalon [7] found that, while loneliness is a significant predictor of cynical hostility, cynical hostility also predicts loneliness. The authors suggested that those with higher levels of cynical hostility may be lacking in social skills and their hostile attributes push others away, or that the cognitive schema ascribing to others a possible hazard deters them from seeking close social relationships. In a series of five studies, including experimental settings, Stavrova et al. [10] found that cynicism and disrespect from others fuel one another in what they have labeled a “vicious cycle.” Those with higher levels of cynical hostility tend to treat others with greater disrespect, which in turn, elicits disrespect toward them. The results of other studies suggest that not only do those with higher levels of cynical hostility perceive less support to be available to them, but they also manage to benefit from it less than their less hostile counterparts [8,25] and are distressed when asked to provide support to others [25].

Most studies that were focused on close social relationships in adulthood examined the role of cynical hostility within the couple dyad. In a study following young individuals over a period of 11 years, it was found that those with higher levels of cynical hostility were more likely be divorced, separated, or in prolonged singlehood [26]. Cynical hostility was also found to be negatively associated with poorer marital adjustment and lower marital quality [27].

Studies that have examined cynical hostility in the context of parent–child relationships have all focused on young children, adolescents, or young adults. Findings of retrospective studies suggest that individuals with higher levels of cynical hostility tend to describe their families of origin as less cohesive, less supportive, and as having lower levels of warmth and greater rejection [28,29]. In a short-term longitudinal study that followed families for two months, parental hostility was predictive of a child’s aggression and conduct problems [30]. In longer term longitudinal studies that followed young children for 12 years through pre-adolescence, mothers’ hostile child-rearing practices were predictive of the later development of their child’s hostility [31].

These studies suggest that parents’ hostility should have short and long-term effects on their children and are in line with Belsky’s [20] theory about the role of personality in parental behavior. Moreover, given that high levels of cynical hostility are related to poorer social relations, it is reasonable to assume that this is another aspect in the lives of highly cynical parents and children that may be adversely impacted, leaving the family without adequate external social support. Given the consistent findings concerning the adverse effect of cynical hostility on individuals’ social relationships in general [7,10] and on close social relationships in particular [9,27], in this paper it is suggested that cynical hostility should also affect the parent–adult child relationship.

When considering the parent–adult child relations, gender differences should also be taken into account. The mother–daughter relationship is considered the most meaningful and closest among parent–child relations, with adult children reporting that they are closer to their mothers than to their fathers [32]. While the mother–daughter relationship is more intimate than any other parent–child dyad [33], it is also more conflictual [34]. Mothers tend to be more expressive of their needs and receive more care from their offspring, especially if they have daughters [35]. Hence, it is possible that mother–child relationships are more resistant to the mother’s cynical hostility than father–child relationships. Focusing on cynical hostility in couples, Segel-Karpas and Ermer [9] found that, while a wife’s loneliness was associated only with her own cynical hostility, her husband’s loneliness was predicted by both his own and his wife’s cynical hostility, suggesting that men are socially more vulnerable to their spouse’s cynical hostility.

In this study, three aspects in the parent–child relationship were examined: strain, support, and contact frequency. These constructs represent qualitative (support and strain) and quantitative (contact) dimensions of relationships and both merit examination, as the results of research suggest that, while children often remain in contact with their parents and provide support if needed, the relationship itself is not always a source of enjoyment or pleasure to the child [13]. Hence, while strain and support may be more adversely affected by a parent’s attributes, contact—especially with mothers—should be relatively resistant, especially in times of need.

To summarize, as previous studies found that not only their own, but also their partner’s, cynical hostility can harm individuals’ social relationships [7,9], it is hypothesized that husbands and wives’ cynical hostility is associated with qualitative and quantitative aspects of their relationship with their children. Specifically, a more cynically hostile parental environment is likely to increase tensions and strains that can result in the reduced willingness of the child to contact the parents and offer support.

**Hypothesis** **1** **(H1).** *Husband and wife’s cynical hostility are positively associated with strain in the relationship with children.*

**Hypothesis** **2** **(H2).** *Husband and wife’s cynical hostility are negatively associated with support in the relationship with children.*

**Hypothesis** **3** **(H3).** *Husband and wife’s cynical hostility are negatively associated with frequency of contact with the children.*

Given the studies in the literature that suggest that asymmetry exists in the relations between mothers and fathers and their children, this research also examined two questions pertaining to differences between husbands’ and wives’ cynical hostility:

Q1: Are there differences between a husband’s and a wife’s cynical hostility and its association with the relationship with children? In other words, do the patterns of association between an individual’s own and his or her spouse’s cynical hostility and the perceived relationship with the children differ between men and women?

Q2: Are there differences in the associations between cynical hostility and the different aspects of relationships (that is, strain, support, and contact)? That is, are there differences between the effects of cynical hostility on the qualitative (strain and support) and quantitative (contact) aspects of the relationship?

To answer these questions and test the hypotheses, two waves of data were used to allow the measurement of residual change. That is, the longitudinal associations between parental hostility and changes in the relationship with the child were tested. Although this design does not preclude alternative explanations of causality, it improves our ability to discuss possible directionality of effect.

## 2. Method

### 2.1. Sample

Data were derived from two waves (T1 and T2) of the Health and Retirement Study (HRS). The HRS is a nationally representative study of the older adult population in the US, with data being collected from individuals aged 50 years and more using a national area probability sampling method of US households. The spouses of eligible participants were also interviewed, regardless of their age, and also completed the full survey questionnaire. The first wave of data was collected in 1992, and data were collected at two-year intervals thereafter, with new cohorts being added to preserve the representative nature of the data of the older population. Since 2006, the HRS added a psychosocial questionnaire (the “leave behind”), which is distributed to a randomly selected half-sample in every other wave, such that longitudinal data are available at four-year intervals. After completion of a face-to-face interview, respondents were asked to fill out the “leave behind” questionnaire and mail it back to the HRS offices. This practice allows greater privacy when answering personal questions. The study was approved by the University of Michigan Institutional Review Board (IRB), and participants were required to sign an informed consent form prior to their participation. For full details on sampling and procedure, please see https://hrs.isr.umich.edu/ (accessed on 27 February 2023).

Cynical hostility was measured only twice for each subsample: in 2006 and 2010, or in 2008 and 2012. For the purpose of this study, data collected in 2006 and 2010, from a sample of 1063 continuously married couples, were used. The response rate was 87.7% in 2006 and 73.1% in 2010 out of the respondents who were eligible to participate in the “leave behind” survey.

### 2.2. Measurements

**Strain** in the relationship with the children was measured using four items rated on a scale ranging from ‘not at all’ (1) to ‘a lot’ (4), each item referring to the respondent’s children. (1.) How often do they make too many demands on you? (2.) How much do they criticize you? (3.) How much do they let you down when you are counting on them? (4.) How much do they get on your nerves? Items were averaged to generate a scale (α = 0.764 and α = 0.779 for husbands and wives, respectively, in 2006, and α = 0.746 and α = 0.761 in 2010).

**Perceived support** from children was measured using three items, similarly rated on a scale ranging from 1 to 4, where each item refers to the respondent’s children: (1.) How much do they really understand the way you feel about things? (2.) How much can you rely on them if you have a serious problem? (3.) How much can you open up to them if you need to talk about your worries? Items were averaged to generate the score (α = 0.819 and α = 0.828 for husbands and wives in 2006, respectively; α = 0.816 and α = 0.781 for husbands and wives in 2010, respectively).

**Contact with children** was measured as the sum of contact with respondent’s children via (1.) in-person meet-ups, (2.) phone calls, and (3.) emails or writing. Each item was rated on a scale ranging from 1 ‘less than once a year or never’ to 6 ‘three or more times a week’.

**Cynical hostility** was measured using 5 items derived from the Cook–Medley hostility scale [36]. Items were rated on a scale ranging from 1 ‘strongly disagree’ to 6 ‘strongly agree’: (1.) Most people dislike putting themselves out to help other people, (2.) Most people will use somewhat unfair means to gain profit or an advantage rather than lose it, (3.) No one cares much what happens to you, (4.) I think most people would lie in order to get ahead, (5.) I commonly wonder what hidden reasons another person may have for doing something nice for me. Items were averaged to create the final scores (α = 0.809, α = 0.791 for husbands and wives, respectively, in 2006; α = 0.801, α = 0.790 for husbands and wives, respectively, in 2010).

Covariates included age, number of children, and co-residency status coded as 1—for at least one child living in respondent’s household, and 0—for no co-residing children.

### 2.3. Analytical Strategy

To test the hypotheses regarding the associations between spouses’ cynical hostility and their relationship with their children, an Actor–Partner Interdependence Model (APIM) was used, within the framework of Structural Equation Modeling (SEM). Each T2 dependent variable (that is, strain, support, and contact) was regressed on the same variable at T1, on both partners’ cynical hostility and covariates (age, number of children, and co-residency with child). Correlation between the T1 independent variables and the T2 dependent variables were allowed. Full information maximum likelihood was used to account for missing data. The Mplus [37] code is available at https://osf.io/b4rmj/.

## 3. Results

Descriptive statistics, including means, standard deviations, and correlations between study variables, are presented in Table 1. Age for men ranged between 31 and 94 (M= 65.72, SD = 8), and between 30 and 86 for women (M = 62.57, SD = 8.16). A total of 85 percent of men and 85.8% of women self-identified as white. Forty-eight percent of men and forty-four percent of women reported they had attained higher education, and mean household income was 89,224.45 USD in 2006 and 82,903 USD in 2010. Fifty three men were under 50 years old (31–49) and were married to older women initially sampled for the study; 219 women were under 50 (30–49) married to older men. Participants had on average 3.3 children (SD = 1.84), and 26.6% (N = 864) had at least one child co-residing with them.

For both husband and wife, cynical hostility is positively correlated with each one’s own strain in the relationship with their children (*r* = 0.28, *p* < 0.001; *r* = 0.22, *p* < 0.001, respectively), and negatively correlated with his or her own support (*r* = −0.21, *p* < 0.001; *r* = −0.16, *p* < 0.001, respectively) and contact with the children (*r* = −0.20, *p* < 0.001; *r* = −0.09, *p* < 0.01, respectively). Husbands and wives’ cynical hostility is also positively associated with their partner’s strain in the relationship with the children (r = 0.13, p < 0.001; r = 0.17, p < 0.001, respectively), negatively associated with their partner’s support from children (r = −0.07, p < 0.05; r = −11, p < 0.01), and negatively associated with their partner’s frequency of contact with children (r = −0.12, p < 0.001; r = −0.17, p < 0.001) (Table 1).

The study hypotheses regarding a husband and a wife’s own and marital partner’s cynical hostility and their associations with strain, support, and contact with children were tested using APIM within the SEM framework. The dependent variables at T2 (strain, support, and contact) were regressed on the same variable at T1, on each partner’s own cynical hostility, his or her partner’s cynical hostility, and the covariates (see Table 2). According to the first hypothesis, an individual’s own and partner’s cynical hostility were expected to predict strain in the relationship with the child. The results (STDYX standardization) indicate that both husband and wife’s cynical hostility is significantly associated with the husband’s strain in the relationship with the children (*b* = 0.16, *p* < 0.001; *b* = 0.06, *p* < 0.05). A wife’s cynical hostility is significantly associated with her own strain in the relationship with the children (*b* = 0.09, *p* < 0.001), but her husband’s cynical hostility is not associated with her strain in the relationship with the children (*b* = 0.04, *p* < 0.10), in partial support of H1.

According to H2, a negative association between both spouses’ cynical hostility and support from children would be found. Results suggest that the husband’s perceived support from children is negatively and significantly associated with his own, but not his wife’s, cynical hostility (*b* = −0.07, *p* < 0.05), and a wife’s perceived support is not significantly associated with either her own or her husband’s cynical hostility. Thus, H2 was only partially supported.

Finally, H3 suggested a negative association between both spouses’ cynical hostility and contact with the children. A husband’s cynical hostility is significantly associated with his own and his wife’s contact (*b* = −0.08, *p* < 0.01; *b* = −0.09, *p* < 0.01, respectively), whereas for a wife, her own cynical hostility is unrelated to contact (Table 2). Fit indices suggest a good fit to the data (*χ^2^* = 105.10, *p* < 0.001; *RMSEA* = 0.049, *CFI* = 0.98, *TLI* = 0.95). This set of results complicates answering the research questions. Indeed, it seems that there is an asymmetry between husbands and wives (Q1) and that this asymmetry is differently expressed in all aspects of the relationship (Q2). While a husband’s strain is negatively affected by his spouse’s cynical hostility, a wife’s contact with the children is affected only by her husband’s cynical hostility. Moreover, while a husband’s support is negatively affected by his own cynical hostility, a wife’s perceived support seems to be resilient to both her own and her partner’s cynical hostility.

To examine further whether the associations with cynical hostility differ according to the mode of contact, an additional analysis was conducted in which the aggregated score of contact was replaced by its three composite variables: in-person meetings, phone calls, and contact via writing (see Table S1). The results suggest that a husband’s meeting with the child is associated with his own hostility (*b* = −0.07, se = 0.03, *p* < 0.05), but not with his wife’s hostility (*b* = −0.01, se = 0.03, *p* = ns). A wife’s meetings are not associated with either her own or her partner’s hostility (*b* = −0.04, se = 0.03, *p* = ns; *b* = 0.03, se = 0.03, *p* = ns). Husbands’ phone calls are not associated with either their own (b = −0.01, se = 0.03, *p* = ns) or their partner’s cynical hostility (*b* = 0.01, se = 0.03, *p* = ns), but wives’ phone calls are positively associated with their own (*b* = 0.06, se = 0.03, *p* < *0*.05), but not their partner’s cynical hostility (*b* = 0.001, se = 0.03, *p* = ns). Finally, husbands’ written communication is marginally significantly associated with their own (*b* = −0.05, se = 0.03, *p* < 0.10) but not their partner’s cynical hostility (*b* = 0.04, se = 0.05, *p* = 0.11). Wives’ written communication is significantly associated with both their own (*b* = −0.06, se = 0.03, *p* < 0.05) and their partner’s (*b* = −0.07, se = 0.03, *p* < *0*.01) cynical hostility.

## 4. Discussion

Given previous findings attesting to the negative social implications [6,7,9,10] of cynical hostility and the continuous importance of the parent–child relationship throughout life, the goal of the current study was to examine the associations between couples’ cynical hostility and their relationship with their adult children, measured in terms of strain, support, and contact.

The findings of the current study suggest that, indeed, parents’ cynical hostility adversely affects their relationship with their adult children and strain seems to be the most susceptible to both the husband’s and the wife’s cynical hostility. Findings indicate that both spouses’ own cynical hostility is positively associated with strain in the relationship with their children. For husbands, their spouse’s cynical hostility is also positively associated with their experienced strain. The children may negatively respond to the parent’s expression of hostile perception, and, possibly, react with disrespect or hostility [10], thus contributing to unpleasant social interactions. The negative effect of both spouses could suggest that a “hostile” parental atmosphere reverberates from one spouse to another, coloring the experience of both with the children. In other words, a hostile parent creates a cascade of negative experiences that prevents the other parent from establishing satisfying relationships with the children [38].

The findings for the perceived support from a child portray an interesting picture. Whereas for husbands there was a negative association between their own cynical hostility and support from a child, no such effect was found for wives. It is surprising, given previous studies suggesting that those having a higher level of cynical hostility tend to perceive lower levels of social support [25]. However, previous studies did not concern the parent–adult child relations. A possible explanation can be drawn from the fathering vulnerability hypothesis [39], suggesting that the father–child bond is more susceptible to marital discord than the mother–child bond. This is in line with findings concerning parental cynical hostility and the father–child bond in younger children [38], and with findings suggesting that mothers are more expressive of their needs and usually enjoy greater support from their offspring [35].

Whereas strain and support are qualitative aspects of relationships, reflecting parents’ subjective evaluation of the interactions, contact is a quantitative aspect suggesting more or less frequent communication. Both a husband and wife’s contact with the children is adversely associated with the husband’s cynical hostility. A wife’s cynical hostility, on the other hand, is unrelated to her own or her husband’s contact. This finding is in some contradiction to the fathering vulnerability hypothesis, which would suggest that a father’s contact with his children should be at greater risk as a result of his own and his spouse’s cynical hostility. A possible explanation may be that husbands’ cynical hostility is expressed in terms of aggression, which deters the children more than the cognitive aspect of cynical hostility. It is also possible that children feel more obligated to contact their mother, rather than their father, and they continue to do so, even if they find her cynical hostility difficult to accept. However, if the father’s hostility dominates the family climate, they may reduce contact nevertheless.

In older adulthood, the costs of problematic relationships can be especially harmful. First, strain in the relationship can have implications for health because of overactivation of the physiological stress responses, making older parents more susceptible to cardiovascular diseases [24,40]. Second, a parent’s cynical hostility may undermine his or her ability to benefit from the child’s offers for support [41]: parents with higher levels of cynical hostility may expect their children to care for them, and at the same time, either reject them or express disbelief in their children’s good intentions (“You only visit because you have to, not because you want to see your old mother”). A hostile communication style can also be a main source of conflict between parents and children . Children may want to gain distance from cynical parents, as the expression of cynical attitudes is aversive to others (that can include not only the child, but also the child’s spouse and offspring). It should also be taken into account that it is possible that these cognitions were expressed during their childhood, making the domestic atmosphere tense and unpleasant, and encouraging them, as adults, to gain distance from their parents [17], for their own sake and the sake of the child’s new founded family. From the parents’ side, it is possible that the cognitions held toward “others” (that is, others are untrustworthy and a source of wrongdoing) are expanded to include the adult children themselves (or their spouses and friends), decreasing the parents’ motivation to interact with their adult child. It is also possible that the adult children also hold cynical hostile attitudes, transmitted via social learning [42], causing both parents and children to be less motivated to interact with one another because of basic distrust in the benefits such interactions can hold. Finally, it is possible that the parent’s cynical hostility leaves him or her isolated from various external sources of support and lacking in a social network [7,9] and thus increases the dependency on a single source of support, that is, the child. This increased dependency and vulnerability may promote a feeling that the relationship is inadequate [43]. This can leave the older parent with unmet needs, which can potentially harm their health and well-being further.

## 5. Limitations and Future Research

The study has several limitations that should be noted. First, the respondents were asked about “the children.” It cannot be determined whether both spouses thought of the same child when replying. It is also possible that the frequency of contact and the extent of support and strain varies between a parent’s different children. As the results of previous studies suggest that the child’s gender plays a role in the parent–child bond in the context of hostility [38], future researchers should collect more specific data, asking parents about their relationship with each child separately. Furthermore, data were collected only from parents, and not from children. As cynical hostility is associated with the perceptions of others’ intentions and behaviors [8], it is possible that the parents who are high in cynical hostility were more inclined to report negative relational experiences with their children. Correspondingly, as previous studies showed that hostility elicits negative reactions from others [10], parents’ hostility might affect the children’s hostility and inclination to interact with others. Thus, future researchers should collect data reported by the child, assessing not only the child’s cynical hostility, but also the child’s perception of provision to and receipt of support from the parent. It is necessary to consider that the data for this research were collected prior to the COVID-19 pandemic. As studies showed that parent–child relations were affected by the pandemic [44,45], researchers should examine the possible effect of cynical hostility on parent–child relations in the unique context of current global changes. It is also important to consider that this study did not differentiate between biological parents to other forms of parent–child relations (such as adoptive families). Future research could examine the long-term effects of cynical hostility in biological vs. other forms of families. Finally, it is possible that strain in the relationship with the child affects the parent’s cynical hostility, or that there is a cycle of hostility, strain, and further hostility. This was not tested in the current study, but could be further explored, especially with data provided by both child and parent.

An examination of the different forms of contact implies that indeed highly hostile fathers meet and exchange written communication with their children less than their less hostile counterparts. Interestingly, higher levels of cynical hostility in mothers and their spouses reduce the mothers’ written communication with their children but increase their contact via phone calls. Given that written communication is quite common, it is possible that this is a prominent indicator of day-to-day communication between parents and their adult children. However, future research is needed to understand further the mechanisms that shape the form of communication between adult children and their parents and the manner in which these are affected by both parent and child attributes.

## 6. Clinical Implications

Cynical hostility, as a cognitive schema, could be subjected to cognitive–behavioral programs aimed at allowing greater flexibility in cognitive patterns. Drawing from the literature concerning aggressive and delinquent men and children, it seems that aggressiveness is the result of attributing ill-intentions to others (a core belief common to cynical hostility). Addressing these cognitive distortions has been a main target of interventions in inmates, for example, and has proven to be effective [46]. For instance, the Growing Pro-Social program (GPS) suggests that aggressive behavior results from a “distorted view of the self and the other” [46, p. 580] and targets cognitive core schemas and distortions. Family practitioners could use the tools offered in the GPS or other cognitive-focused intervention programs to address parents’ (and children’s) cynical hostile beliefs, reducing tensions, disrespect, and distance between adult children and their parents.

## 7. Conclusions

Cynical hostility is a social cognitive schema with adverse effects for individuals’ health, well-being, and social relationships [6]. The findings of the current study contribute to the literature by highlighting the toll that both partners’ cynical hostility takes on their relationship with their adult children in terms of strain, support, and contact. As adult children often provide support to their older parents [18], and as cynical hostility increases vulnerability to health problems [24], older adults having higher levels of cynical hostility may be especially susceptible to lack of adequate support in times of need. Cynical hostility should be explored further as a factor that drives social relationships in general and family relationships in particular, with an emphasis on its implications for health and well-being in older adulthood.

## Figures and Tables

**Table 1:** Descriptive statistics and correlations between main study variables.

	M	SD	1	2	3	4	5	6	7	8	9	10	11	12	13	14	15	16	17
1. H’s age	64.81	8.39																	
2. W’s age	61.49	8.63	0.79 ***																
3. Children (n)	3.28	1.95	0.18 ***	0.14 ***															
4. Co-residency	0.26	0.44	−0.31 ***	−0.35 ***	0.07 ***														
5. H’s hostility	3.13	1.11	−0.08 **	−0.12 ***	0.09 **	0.113 ***													
6. W’s hostility	2.72	1.08	−0.08 **	−0.11 ***	0.07 **	0.096 ***	0.33 ***												
7. H’s strain T1	1.74	0.6	−0.17 ***	−0.15 ***	0.04	0.200 ***	0.25 ***	0.17 ***											
8. W’s strain T1	1.77	0.62	−0.19 ***	−0.21 ***	−0.01	0.254 ***	0.13 ***	0.22 ***	0.43 ***										
9. H’s support T1	0.73	3.14	0.20 ***	0.23 ***	−0.04	−0.068 *	−0.24 ***	−0.13 ***	−0.41 ***	−0.27 ***									
10. W’s support T1	0.68	3.31	0.14 ***	0.19 ***	0.04	−0.065 *	−0.10 ***	−0.20 ***	−0.24 ***	−0.41 ***	0.34 ***								
11. H’s contact T1	11.42	2.95	−0.03	0.04	−0.10 ***	−0.050	−0.15 ***	−0.14 ***	−0.08 **	−0.08 **	0.30 ***	0.14 ***							
12. W’s contact T1	12.33	2.75	−0.01	0.02	−0.02	−0.043	−0.09 **	−0.16 ***	−0.07 *	−0.08 **	0.14 ***	0.30 ***	0.43 ***						
13. H’s strain T2	1.65	0.58	−0.19 ***	−0.20 ***	0	0.190 ***	0.28 ***	0.17 ***	0.55 ***	0.34 ***	−0.28 ***	−0.18 ***	−0.08 **	−0.08 *					
14. W’s strain T2	1.68	0.6	−0.16 ***	−0.19 ***	−0.04	0.171 ***	0.13 ***	0.22 ***	0.34 ***	0.61 ***	−0.18 ***	−0.31 ***	−0.02	−0.05	0.46 ***				
15. H’s support T2	0.74	3.14	0.20 ***	0.24 ***	−0.03	−0.022	−0.21 ***	−0.11 ***	−0.33 ***	−0.20 ***	0.64 ***	0.27 ***	0.28 ***	0.11 ***	−0.36 ***	−0.24 ***			
16. W’s support T2	0.64	3.32	0.0 9**	0.14 ***	0.03	0	−0.07 *	−0.16 ***	−0.18 ***	−0.27 ***	0.22 ***	0.60 ***	0.08 **	0.24 ***	−0.24 ***	−0.38 ***	0.30 ***		
17. H’s contact T2	11.41	3.05	−0.09 **	−0.02	−0.14 ***	−0.046	−0.20 ***	−0.17 ***	−0.08 **	−0.03	0.28 ***	0.09 ***	0.63 ***	0.30 ***	−0.08 **	−0.02	0.34 ***	0.10 **	
18.W’s contact T2	12.67	2.9	−0.13 ***	−0.09 **	−0.01	0.043	−0.12 ***	−0.09 **	−0.02	0	0.07 *	0.22***	0.26 ***	0.53 ***	−0.08 **	−0.02	0.07 *	0.32 ***	0.32 ***

* *p* < 0.05; ** *p* < 0.01; *** *p* < 0.001. H’s = husband’s; W’s = wife’s. Co-residency: 1 = at least one child co-reside with respondent. 0 = no children co-reside with respondent.

**Table 2:** Dyadic analysis of husband and wife strain in their relationships with their children.

	Husband’s Strain with Children		Wife’s Strain with Children		Husband’s Support from Child		Wife’s Support from Child		Husband’s Contact with Children		Wife’s Contact with Children	
	*b*	*se*	*b*	*se*	*b*	*se*	*b*	*se*	*b*	*se*	*b*	*se*
Respondent’s age	−0.10 ***	0.03	−0.05 *	0.03	0.14 ***	0.03	0.05 *	0.03	−0.06 *	0.03	−0.08 ***	0.03
Number of children	0.03	0.03	−0.02	0.03	−0.06 *	0.03	0.02	0.02	−0.07 ***	0.03	0.02	0.03
Co-residency ^1^	0.05	0.03	0.003	0.03	0.05	0.03	0.05 *	0.03	−0.02	0.03	0.03	0.03
Husband’s strain with children T1	0.45	0.02										
Wife’s strain with children T1			0.55	0.02								
Husband’s support from children T1					0.57 ***	0.02						
Wife’s support from children T1							0.58 ***	0.02				
Husband’s contact with children T1									0.59 ***	0.02		
Wife’s contact with children T1											0.53 ***	0.02
Husband’s hostility	0.16 ***	0.03	0.04	0.03	−0.07 *	0.02	−0.006	0.01	−0.08 **	0.03	−0.09 **	0.03
Wife’s hostility	0.06 *	0.03	0.09 ***	0.03	−0.01	0.02	−0.03	0.01	−0.03	0.03	0.01	0.03

* *p* < 0.05; ** *p* < 0.01; *** *p* < 0.001. 1 = Children co-residing, 0 = no co-residing children.

## Data Availability

The data is publicly available at https://hrs.isr.umich.edu/.

## Supplementary Materials

The following are available online at https://www.mdpi.com/article/10.3390/healthcare11050736/s1, Table S1: Dyadic analysis of husband and wife contact with their children via meetings, phone calls, and writing.
