# Supplementary material for: Older Parents’ Cynical Hostility and Their Relationships with Their Adult Children: A Longitudinal Dyadic Study of North American Couples
Source: Healthcare (Basel). 2023 Mar 2;11(5):736. doi: 10.3390/healthcare11050736 (PMC10001209; doi:10.3390/healthcare11050736)
Supplement: Supplementary file 1 [file healthcare-11-00736-s001.zip › healthcare-2052472-supplementary.pdf]

Appendix S1

Table S1: Dyadic analysis of husband and wife contact with their children via meetings, phone calls, and writing

	Husband's contact via meetings		Wife's contact via meetings		Husband's contact via phone calls		Wife's contact via phone calls		Husband's contact via writing		Wife's contact via writing	
	b	*se*	*b*	*se*	*b*	*se*	*b*	*se*	*b*	*se*	*b*	*se*
Respondent's age	0.02	0.03	0.04	0.03	-0.01	0.03	0.01	0.03	-0.08***	0.03	-0.15***	0.03
Number of children	-0.06*	0.03	0.03	0.03	-0.06*	0.03	-0.01	0.03	-0.05	0.03	-0.02	0.03
Co-residency[1]	0.001	0.03	0.06	0.03	0.001	0.03	0.07*	0.03	-0.04	0.03	0.01	0.03
T1 dependent var[2]	0.53***	0.02	0.51***	0.02	0.50***	0.02	0.53***	0.02	0.62***	0.02	0.61***	0.03
Husband's hostility	-0.07*	0.03	-0.05	0.03	-0.01	0.03	-0.001	0.03	-0.05+	0.03	-0.08***	0.03
Wife's hostility	-0.01	0.03	0.03	0.03	0.01	0.03	0.06*	0.03	-0.04	0.03	-0.06*	0.03

+ $p<.10$;   *$p<.05$;   ***$p<.001$

1. 1=Children co-residing; 0=no co-residing children.
2. Each dependent variable was regressed on the same variable at T1.
